# Pirfenidone anti-fibrotic effects are partially mediated by the inhibition of MUC1 bioactivation

**DOI:** 10.18632/oncotarget.27526

**Published:** 2020-04-14

**Authors:** Beatriz Ballester, Javier Milara, Julio Cortijo

**Affiliations:** ^1^Department of Pharmacology, Faculty of Medicine, University of Valencia, Valencia, Spain; ^2^CIBERES, Health Institute Carlos III, Valencia, Spain; ^3^Health Research Institute INCLIVA, Valencia, Spain; ^4^Pharmacy Unit, Clinic University Hospital, Valencia, Spain; ^5^Research and Teaching Unit, University General Hospital Consortium, Valencia, Spain; ^*^These authors contributed equally to this work

**Keywords:** pirfenidone, idiopathic pulmonary fibrosis, MUC1, transforming growth factor beta-1, fibroblasts

## Abstract

Pirfenidone is a pleiotropic molecule approved to treat idiopathic pulmonary fibrosis (IPF). Pirfenidone has demonstrated to downregulate transforming growth factor-β1 (TGF-β1) cellular effects. However, its anti-fibrotic mechanism remains unclear. Here, we aim to analyze the effects of pirfenidone on the TGF-β1 canonical and non-canonical pathways, as well as, on the most characteristic IPF cellular processes. Results observed in this work showed that TGF-β1-induced canonical SMAD3 and non-canonical ERK1/2 phosphorylations were not inhibited by pirfenidone in alveolar A549 and lung fibroblasts MRC5 cells. In contrast, pirfenidone inhibited TGF-β1-induced MUC1-CT Thr^41^ (1224) and Tyr^46^ (1229) phosphorylations, thus reducing the β-catenin activation. Additionally, immunoprecipitation and immunofluorescence studies in ATII cells and lung fibroblasts showed that pirfenidone inhibited the formation and nuclear translocation of the transcriptional fibrotic TGF-β1-induced phospho-SMAD3/MUC1-CT/active-β-catenin complex, and consequently the SMAD-binding element activation (SBE). This study provided also evidence of the inhibitory effect of pirfenidone on the TGF-β1-induced ATII to mesenchymal and fibroblast to myofibroblast transitions, fibroblast proliferation and ATII and fibroblast senescence. Therefore, it indicates that pirfenidone’s inhibitory effect on TGF-β1-induced fibrotic cellular processes is mediated by the inhibition of MUC1-CT phosphorylation, β-catenin activation, nuclear complex formation of phospho-SMAD3/MUC1-CT/active β-catenin and SBE activation, which may be of value to further develop anti-fibrotic IPF therapies.

## INTRODUCTION

Idiopathic pulmonary fibrosis (IPF) is a devastating disease characterized by extensive accumulation of abnormal extracellular matrix (ECM) in the lung and variable progression among patients, leading to death 3-5 years after diagnosis [1]. IPF is currently seen as the consequence of an unresolved wound healing [2], which is mediated by several pro-fibrotic factors and leads to an uncontrolled proliferation of myofibroblasts, responsible for the abnormal ECM production. Myofibroblasts are key cells of the fibrotic disease and they are characterized by the expression of alpha-smooth muscle actin (α-SMA) and migration capacities [3]. Invasive myofibroblasts in IPF lungs have multiple origins [4] including lung resident fibroblasts or mesenchymal/myofibroblast transformations of alveolar type II epithelial cells (ATII) [5]. Furthermore, both type of cells, fibroblasts and ATII cells, acquire senescent identities, which are able to promote lung fibrosis [6, 7].

Transforming growth factor (TGF)-β1 is probably the most potent pro-fibrotic factor [8]. This cytokine is known to promote fibroblasts differentiation and ECM production [9], leading to pulmonary fibrosis mainly through the SMAD-dependent canonical pathway [10]. Nevertheless, TGF-β1 also propagates its signalling by the SMAD-independent non-canonical pathway, through other factors such as, p38 mitogen-activated protein kinase (p38 MAPK), phosphoinositide 3-kinase (PI3K), β-catenin, AKT (also known as protein kinase B), extracellular signal-regulated kinase (ERK), and JUN N-terminal kinase (JNK) among others [8]. Indeed, aberrant activity of the developmental Wnt/β-catenin signaling pathway has also emerged recently as a fundamental concept in fibrogenesis [11, 12].

Transmembrane mucin 1 (MUC1) is a large glycoprotein that acts as a membrane receptor. It is comprised of a extracellular tandem repeat domain, a transmembrane domain, and a C-terminal cytoplasmic tail (CT), which contains several potential and putative tyrosine or serine/threonine phosphorylation sites [13]. MUC1 extracellular domain contains the KL6 epitope domain, which can be shed into the lumen by proteolytic cleavage [14] and it has been observed increased in serum, broncho-alveolar lavage fluid (BALF) and lung tissue of IPF patients [13], serving as a potential biomarker in IPF disease [15]. Otherwise, the highly conserved CT of MUC1 modulates multiple intracellular signals [16] by phosphorylation, bioactivation and interaction with several proteins implicated in different carcinogenic processes linked to IPF disease [17, 18]. In this context, we found recently that the expression of MUC1-CT and its phosphorylated forms at Thr^41^ and Tyr^46^ were increased in lung tissue from IPF patients. We also observed that TGF-β1, through SMAD3 phosphorylation, increased the intracellular bioactivation of MUC1-CT, thus increasing the expression of the active β-catenin to form a nuclear complex of phospho-SMAD3/MUC1-CT and MUC1-CT/β-catenin, promoting the development of fibrotic processes such as ATII and fibroblast to myofibroblast transitions, as well as, cell senescence and fibroblast proliferation [19].

Currently, therapeutic option to treat IPF is still limited. Indeed, pirfenidone, which has been recently approved as IPF therapy [20], demonstrates only a reduction of the loss of lung function, but is not able to reverse IPF progression. Pirfenidone, is a pleiotropic molecule that has anti-oxidant, anti-fibrotic and anti-inflammatory properties as shown in several *in vitro* and *in vivo* studies [21, 22]. As an anti-fibrotic therapy, it has been demonstrated that pirfenidone inhibits TGF-β-induced cellular processes, collagen synthesis, fibroblast proliferation and ATII fibrotic gene expression, as well as, mediates tissue repair [23–27]. However, the exact mechanisms through which pirfenidone offers protection against lung fibrosis remains unclear.

In this study, we analysed *in vitro* the anti-fibrotic mechanism of pirfenidone on the TGF-β1 canonical and non-canonical pathways as well as on the effects of cellular tranformations such as ATII and fibroblast to myfibroblast transitions, cell senescence and fibroblast proliferation.

## RESULTS

### Pirfenidone suppresses TGF-β1 non-canonical β-catenin activation but not non-canonical ERK1/2 and canonical SMAD3 phosphorylation

**Figure 1:**
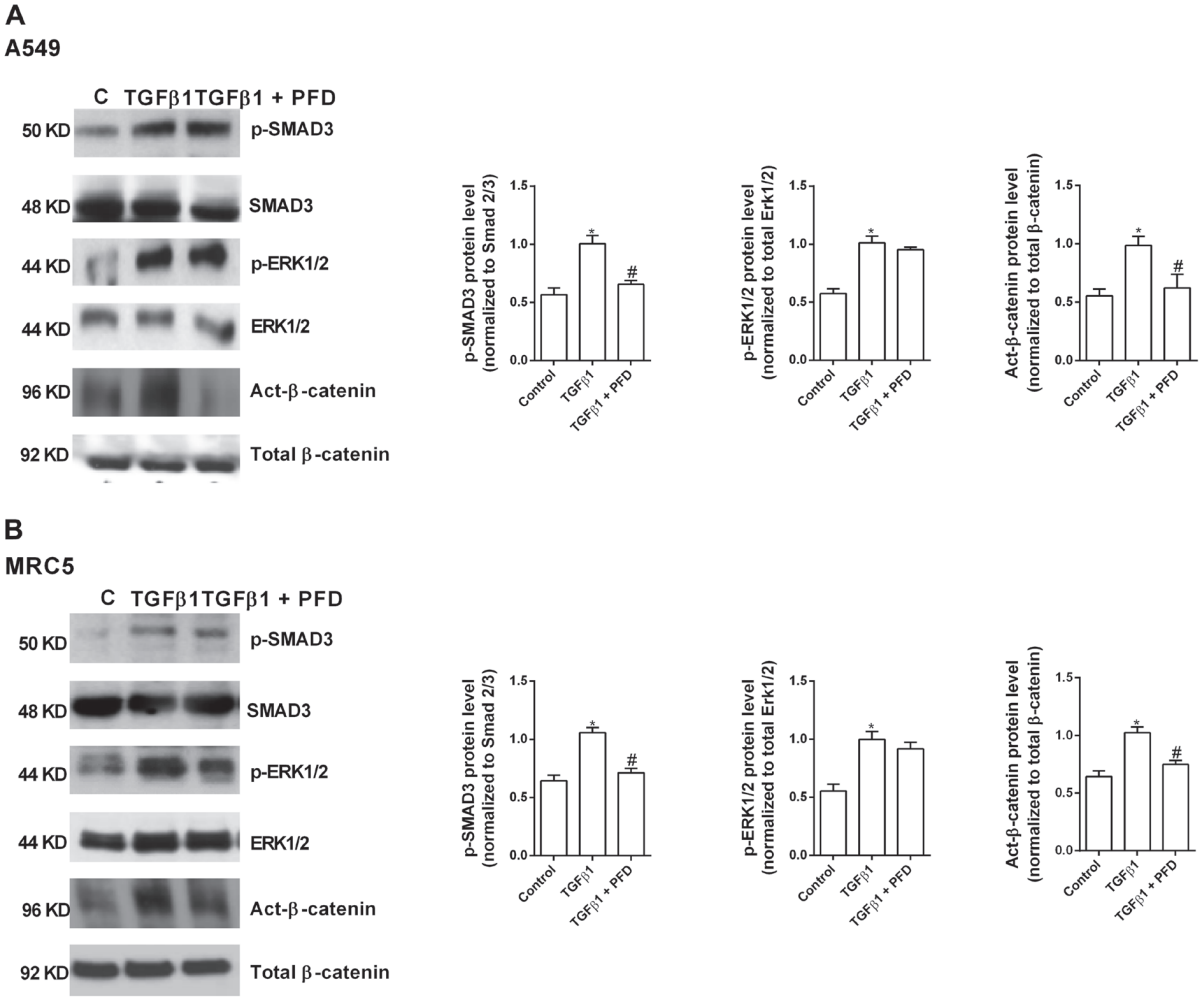
Pirfenidone (PFD) inhibits the TGF-β1-induced β-catenin activation but not the SMAD3 and ERK1/2 phosphorylation. A549 (**A**) and MRC5 (**B**) cells were stimulated 40 min with TGFβ1 5 ng/ml in the presence or absence of PFD 50 µM. Total protein was analyzed by western blot and quantified by densitometry. Protein expression of phospho (p)-SMAD3, p-ERK1/2 and active (act)-β-catenin was measured. Data are expressed as the ratio to total Smad3, total ERK1/2 or total β-catenin protein. Sample Western blots from a single representative experiment are shown. One-way ANOVA was followed by the post hoc Bonferroni test. ^*^ *P* < 0.05 vs. control; ^#^ *P* < 0.05 vs. TGFβ1.

The canonical SMAD3 phosphorylation was increased in A549 cells by TGF-β1 action (Figure 1A). Pirfenidone did not prevent this SMAD3 phosphorylation (Figure 1A). Similar results were observed in MRC5 lung fibroblast cell line (Figure 1B) in which pirfenidone did not prevent the TGF-β1-induced SMAD3 phosphorylation.

The non-canonical β-catenin activation pathway was also increased in A549 and MRC5 cells by TGF-β1 action (Figure 1A) and inhibited by pirfenidone (Figure 1A, 1B). The non-canonical ERK1/2 phosphorylation pathway was also increased in A549 and MRC5 cells by TGF-β1 action, although similarly to SMAD3 phosphorylation, pirfenidone did not prevent ERK1/2 phosphorylation in both types of cells (Figure 1A, 1B).

### Pirfenidone inhibits TGF-β1-induced MUC1-CT bioactivation

**Figure 2:**
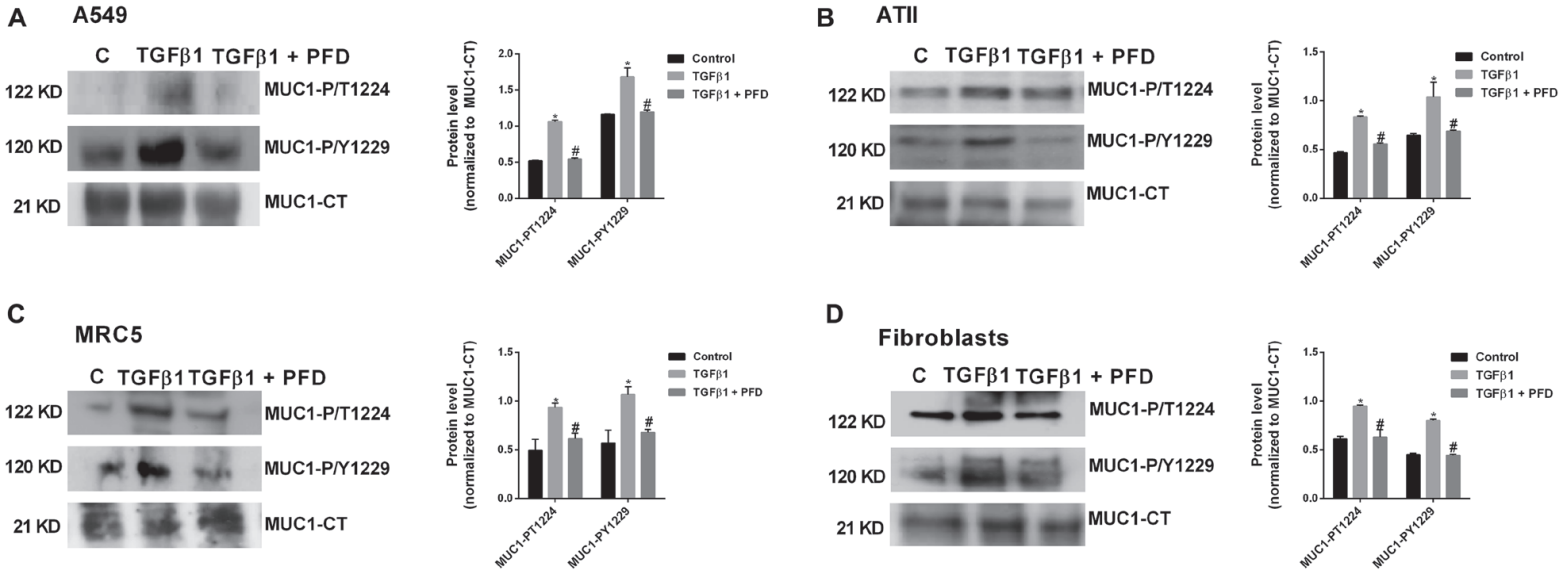
Pirfenidone (PFD) supresses the TGF-β1-induced MUC1-cytoplasmic tail (CT) bioactivation: ATII cells were isolated from the lungs of control subjects, and lung fibroblasts were isolated from the lungs of IPF patients. A549 cells (**A**), ATII cells (**B**), primary lung fibroblasts (**C**) and MRC5 cells (**D**) were stimulated 40’ with TGFβ1 5 ng/ml in the presence or absence of PFD 50 µM. Total protein was analyzed by western blot and quantified by densitometry. Protein expression of MUC1-CT, MUC1-P/T-1224 and MUC1-P/Y-1229 (A–D) and active (act)-β-catenin and total β-catenin (A, C) was analysed by western blot. Data are expressed as the ratio to MUC1-CT or total β-catenin protein. Results are expressed as mean ± SE of *n* = 3 independent experiments run in triplicate. Sample Western blots from a single representative experiment are shown. One-way ANOVA (for A549 or MRC5 cells) or two-way ANOVA (for primary ATII and lung fibroblasts) followed by post-hoc bonferroni tests. ^*^ *P* < 0.05 vs. control; ^#^ *P* < 0.05 vs. TGFβ1.

TGF-β1 significantly increased the MUC1-CT phosphorylation at the 1224 threonine and 1229 tyrosine amino acid positions after 40 min of stimulation in A549 (Figure 2A) and MRC5 cells (Figure 2C). In contrast, in cells incubated with pirfenidone, TGF-β1 was not able to induce these MUC1-CT phosphorylations. Similar results were observed in primary ATII cells (Figure 2B) and primary lung fibroblasts (Figure 2D). TGF-β1-induced MUC1-CT phosphorylations at the 1224 threonine and 1229 tyrosine amino acid positions were inhibited by pirfenidone.

### Pirfenidone inhibits the TGF-β1-induced phospho-Smad3/MUC1-CT and MUC1-CT/active-β-catenin nuclear protein complex and Smad-binding element activation

**Figure 3:**
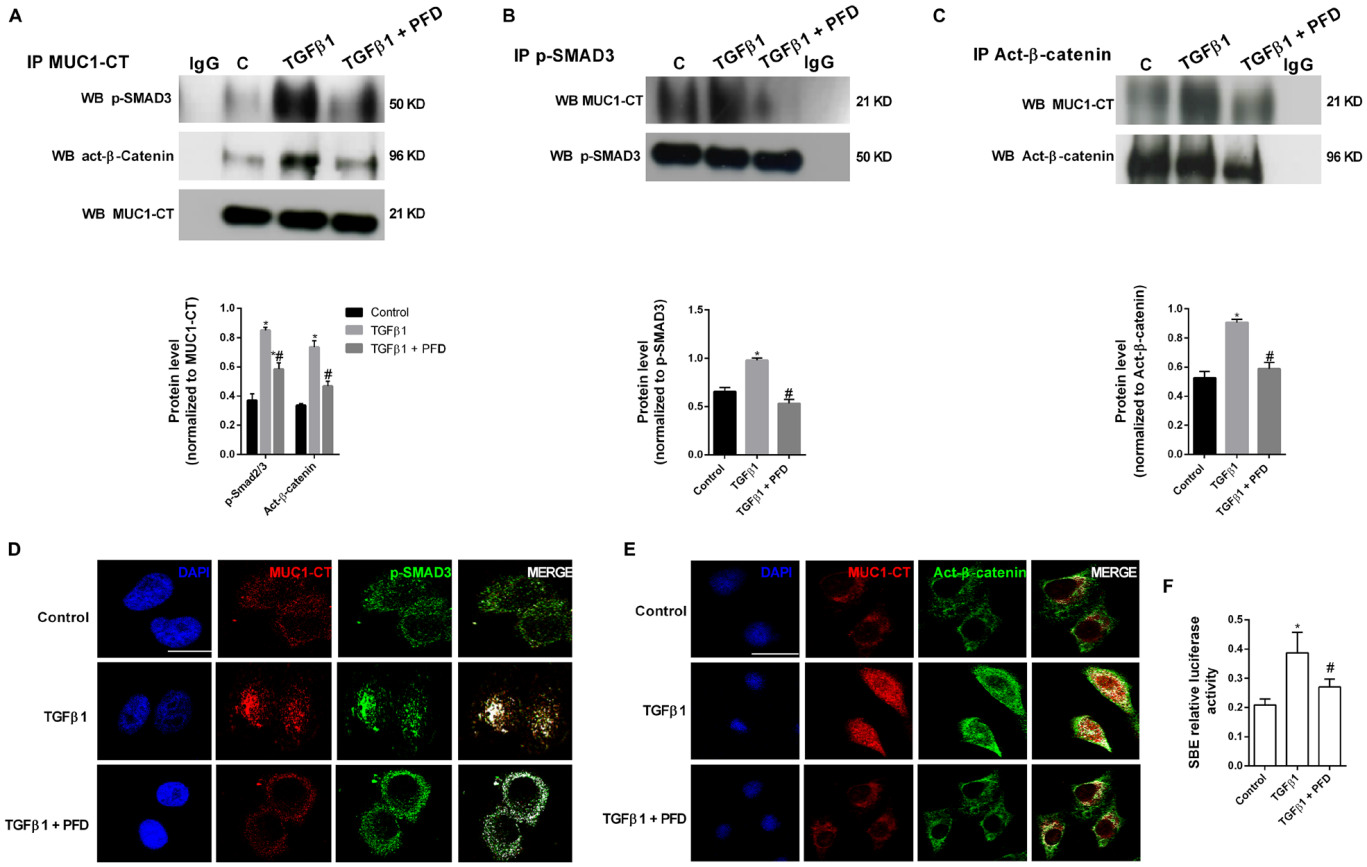
Pirfenidone (PFD) inhibits MUC1-CT co-localization with phospho (p)-Smad3 and active (act)-β-catenin in the nuclei of alveolar epithelial type II (ATII) cells stimulated with TGFβ1. (**A**–**E**) ATII cells were stimulated with TGFβ1 5 ng/ml for 1h in the presence or absence of PFD 50 µM. (A–C) Total protein was extracted and immunoprecipitated with MUC1-CT, phospho (p)-SMAD3 or active (act)-β-catenin. Different western blots were proved against active (act)-β-catenin, phospho-SMAD3 and MUC1-CT. Non-specific IgG was used as a negative isotype control for immunoprecipitation. Sample Western blots from a single representative experiment are shown. (D, E) Co-localization was analyzed using a con-focal spectral microscope (Leica TCS SP2) that generated a bidimensional cytofluorogram that selected common localized points of both antibodies (white color). Scale bars: 10 μm. Con-focal immunofluorescence microscope images showed the nuclear translocation and co-localization of MUC1-CT/p-SMAD3 and MUC1-CT/act-β-catenin, which was inhibited by PFD. (**F**) A549 cells were stimulated 18 h with TGFβ1 10 ng/ml in the presence or absence of PFD 50 µM. Activation of the Smad binding element (SBE) was measured. Results are expressed as mean ± SE of *n* = 3 independent experiments. One-way ANOVA (for A549 cells) or two-way ANOVA (for primary ATII cells) followed by post-hoc bonferroni tests. ^*^ *P* < 0.05 vs. control; ^#^ *P* < 0.05 vs. TGFβ1.

**Figure 4:**
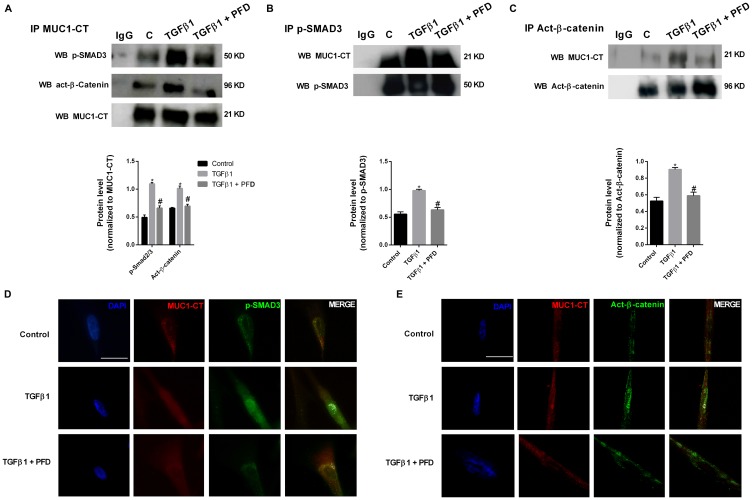
Pirfenidone (PFD) inhibits MUC1-CT co-localization with pSmad3 and active-β-catenin in the nuclei of lung fibroblasts stimulated with TGFβ1. Primary lung fibroblasts from IPF patients were stimulated with TGFβ1 5 ng/ml for 1 h in the presence or absence of pirfenidone (PFD) 50 µM. (**A**–**C**) Total protein was extracted and immunoprecipitated with MUC1-CT, phospho (p)-SMAD3 or active (act)-β-catenin. (A–C) Different western blots were proved against active (act)-β-catenin, phospho-SMAD3 and MUC1-CT. Non-specific IgG was used as a negative isotype control for immunoprecipitation. Results are expressed as mean ± SE of *n* = 3 independent experiments. Sample Western blots from a single representative experiment are shown. Two-way ANOVA followed by post-hoc bonferroni tests. ^*^ *P* < 0.05 vs. control; ^#^ *P* < 0.05 vs. TGFβ1. (**D**, **E**) Co-localization was analyzed using a con-focal spectral microscope (Leica TCS SP2) that generated a bidimensional cytofluorogram that selected common localized points of both antibodies (white color). Scale bars: 10 μm. Con-focal immunofluorescence microscope images showed the nuclear translocation and co-localization of MUC1-CT/p-SMAD3 and MUC1-CT/act-β-catenin, which was inhibited by PFD.

In ATII cells, TGF-β1 stimulus promoted the formation of protein complexes including phospho-SMAD3/MUC1-CT and active (act)-β-catenin/MUC1-CT as showed the immunoprecipitations (Figure 3A, 3B, 3C) and confocal microscopy studies (Figure 3D, 3E). In A549 cells, TGF-β1 induced Smad-binding element (SBE) activation, which was attenuated by pirfenidone pretreatment (Figure 3F). Therefore, it confirms that preincubation with pirfenidone has little effect on the TGF-β1-induced phosphorylation of SMAD3, but abrogates the TGF-β1-induced nuclear localization of SMAD3. TGF-β1 stimulus also promoted the nuclear protein complexes of phospho-SMAD3/MUC1-CT and act-β-catenin/MUC1-CT in primary lung fibroblast from IPF patients, as showed by immunoprecipitation (Figure 4A–4C) and con-focal immunofluorescence (Figure 4D, 4E). Pirfenidone prevented the formation of these protein complexes, as well as, the nuclear localization of them in IPF primary lung fibroblasts.

### Pirfenidone inhibits the TGF-β1-induced alveolar type II epithelial to mesenchymal and fibroblast to myofibroblast transitions

**Figure 5:**
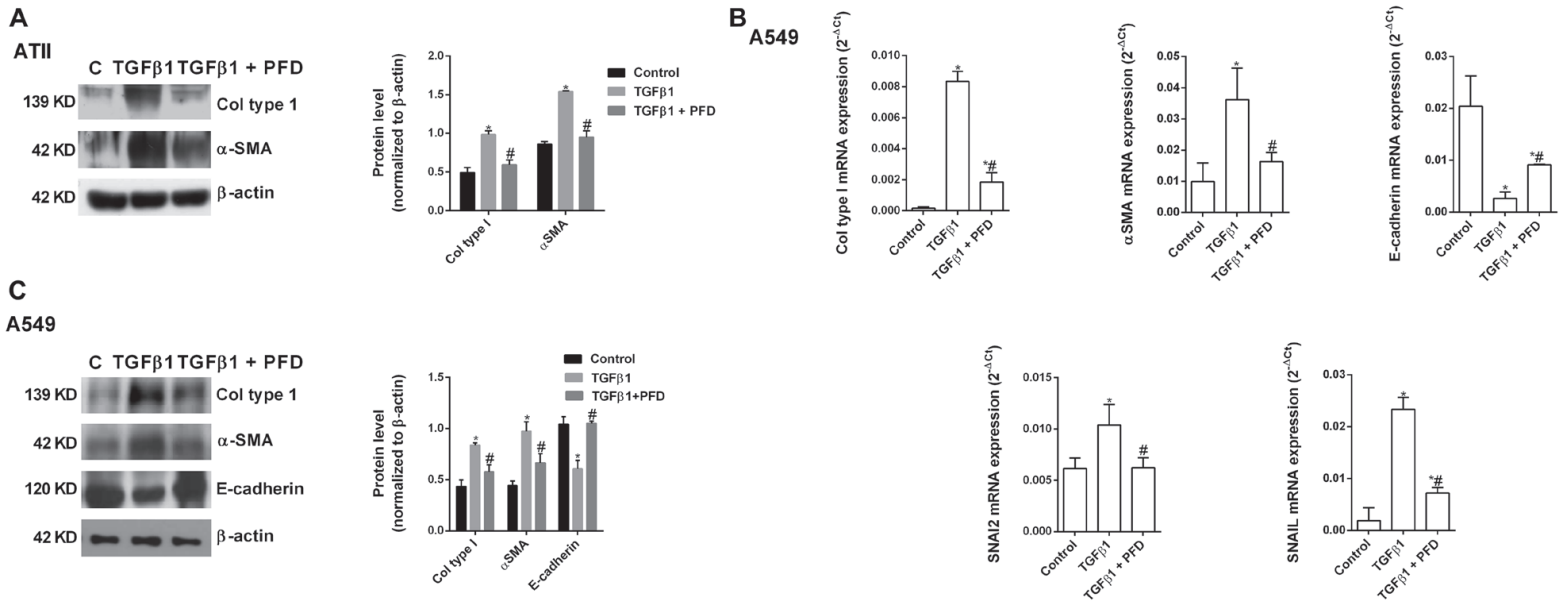
Pirfenidone (PFD) inhibits the TGF-β1-induced alveolar type II (ATII) to mesenchymal transition. Primary ATII cells were isolated from the lungs of control subjects. ATII cells (**A**) or A549 cells (**B**, **C**) were stimulated 72 h (A, B) or 48 h (C) with TGFβ1 5 ng/ml in the presence or absence of PFD 50 µM. Total protein and RNA from cell lysates were analyzed by (A, B) western blot and quantified by densitometry and by (C) qPCR. α-SMA, collagen type I and E-cadherin were measured using (A, B) western blot. Collagen type I, α-SMA, E-cadherin, SNAI2 and SNAIL were measured using (C) RT-PCR. Data are expressed as the ratio to β-actin protein and 2^-∆Ct^ for mRNA levels. The results are expressed as mean ± SE of *n* = 3 independent experiments run in triplicate. Sample Western blots from a single representative experiment are shown. One-way ANOVA (for A549 cells) or two-way ANOVA (primary ATII) followed by post-hoc bonferroni tests. ^*^ *P* < 0.05 vs. control; ^#^ *P* < 0.05 vs. TGFβ1.

**Figure 6:**
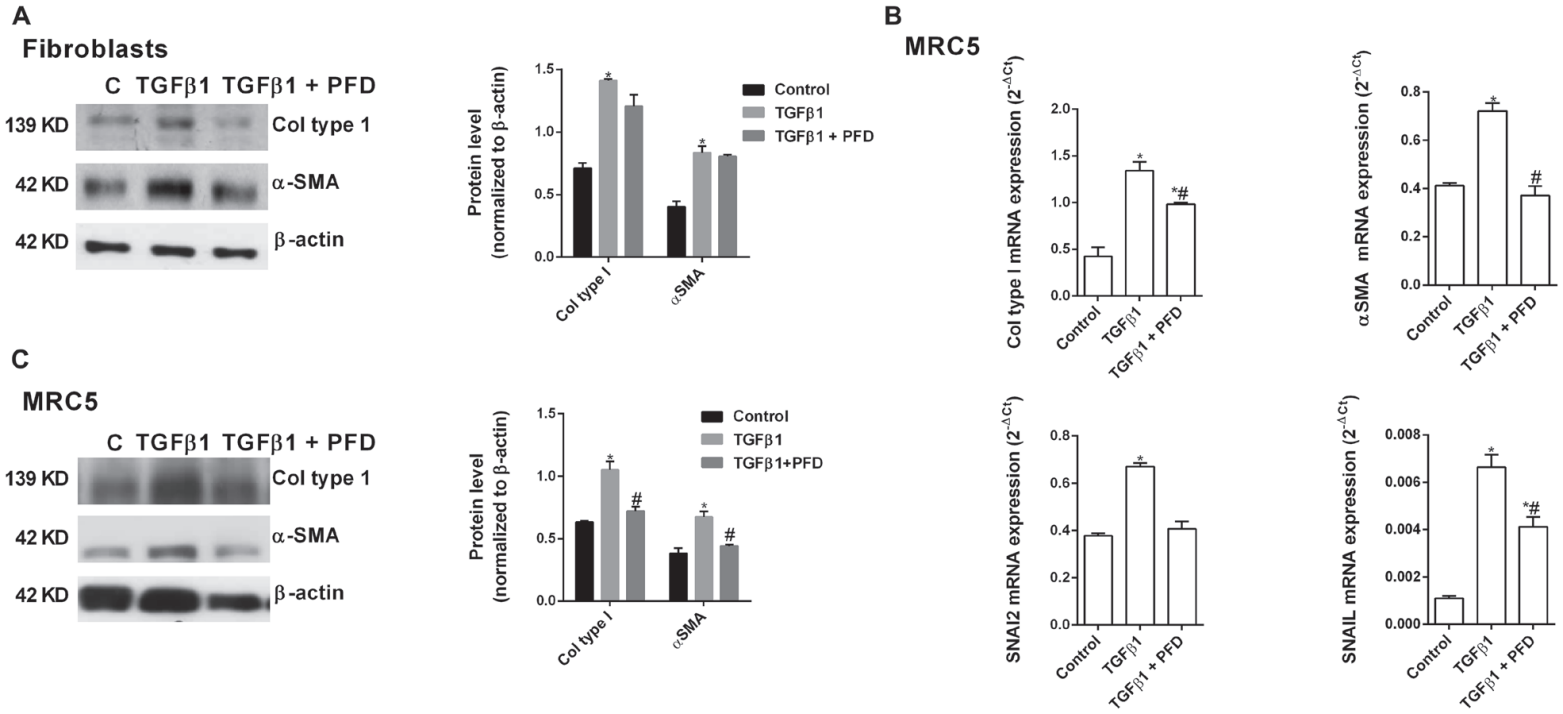
Pirfenidone (PFD) inhibits the TGF-β1-induced fibroblast to myofibroblast transition. Primary lung fibroblasts were isolated from the lungs of IPF patients. Primary lung fibroblasts (**A**) or MRC5 cells (**B**, **C**) were stimulated 72 h (A, B) or 48h (C) with TGFβ1 5 ng/ml in the presence or absence of pirfenidone (PFD) 50 µM. Total protein and RNA from cell lysates were analyzed by (A, B) western blot and quantified by densitometry and by (C) qPCR. α-SMA and collagen type I were measured using (A, B) western blot. Collagen type I, α-SMA, SNAI2 and SNAIL were measured using (C) RT-PCR. Data are expressed as the ratio to β-actin protein and 2^-∆Ct^ for mRNA levels. The results are expressed as mean ± SE of *n* = 3 independent experiments run in triplicate. Sample Western blots from a single representative experiment are shown. One-way ANOVA (for A549 cells) or two-way ANOVA (primary ATII) followed by post-hoc bonferroni tests. ^*^ *P* < 0.05 vs. control; ^#^ *P* < 0.05 vs. TGFβ1.

TGF-β1 promoted the ATII to mesenchymal transition in primary cells (Figure 5A) and the bronchoalveolar cell line A549 (Figure 5B, 5C), increasing the protein and gene expression of the canonical TGF-β1 mesenchymal markers α-SMA, collagen type I, SNAI2 and SNAIL, and decreasing the expression of the epithelial marker E-cadherin after 72 h of stimulation. Pirfenidone inhibited ATII to mesenchymal transition since pirfenidone inhibited the canonical TGF-β1-induced expression of mesenchymal markers, as well as, the TGF-β1-induced decrease of E-cadherin expression. Similar results were observed in primary lung fibroblasts (Figure 6A) and MRC5 lung fibroblast cell line (Figure 6B, 6C). TGF-β1 induced fibroblast to myofibroblast transition (Figure 6A–6C), which was inhibited by pirfenidone treatment. Nevertheless, pirfenidone’s inhibitory action of pirfenidone on TGF-β1-induced transition of primary fibroblasts to myofibroblasts was not reported as statistically significant, which is just probably due to the small pirfenidone concentrations used in this study.

### Pirfenidone attenuates the TGF-β1-induced ATII and lung fibroblast senescence and the TGF-β1-induced lung fibroblast proliferation

**Figure 7:**
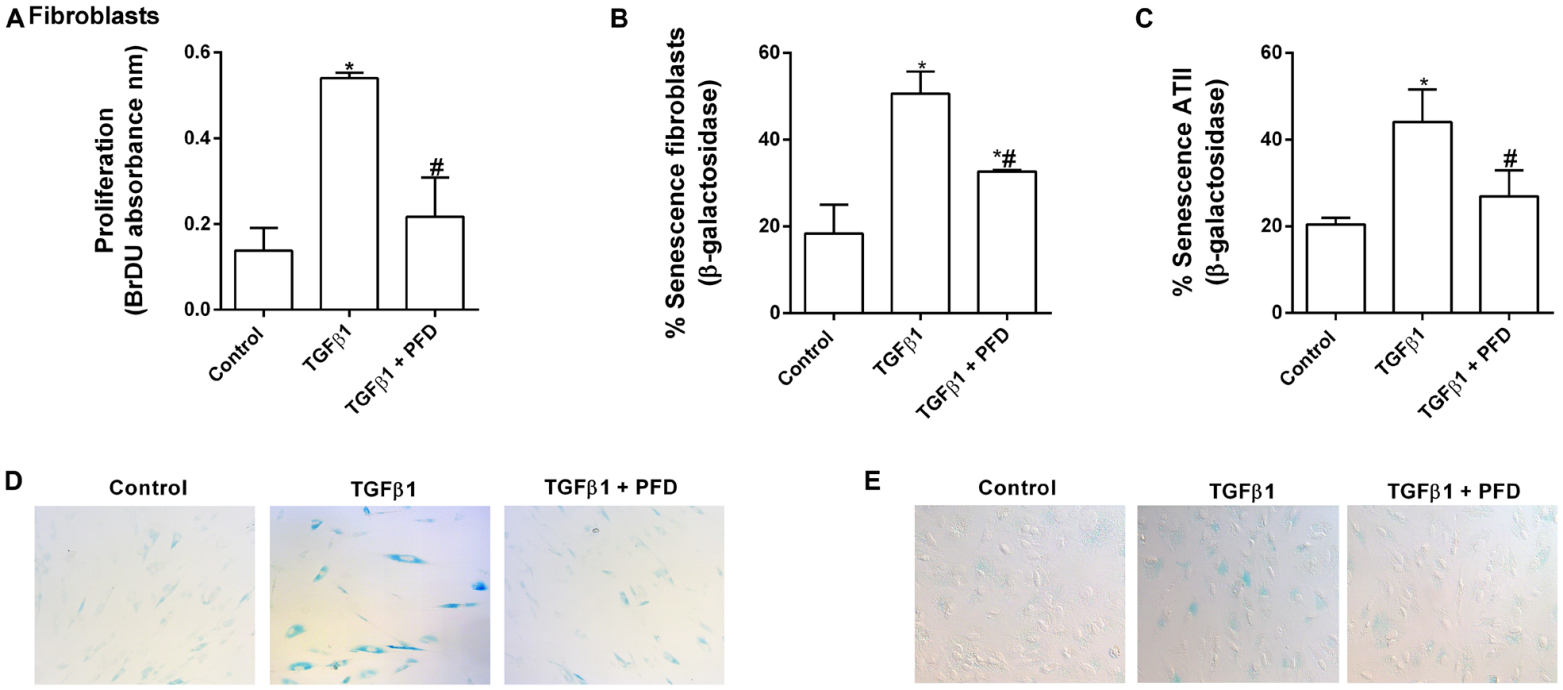
Pirfenidone (PFD) attenuates TGF-β1-induced lung fibroblast proliferation and senescence of epithelial alveolar type II (ATII) cells and fibroblasts. ATII cells were isolated from the lungs of control subjects, and lung fibroblasts were isolated from the lungs of IPF patients. (**A**) Fibroblast proliferation during 48 h was evaluated by the BrdU assay. PFD (50 µM) was added 30 min before 10% foetal bovine serum and 10 ng/ml TGFβ1. (**D**) Primary human lung fibroblasts and (**E**) primary human ATII cells were stained for senescence-associated β-galactosidase activity and microscopic (magnification of 40×) images were taken. (**B**, **C**) Percentage of cells expressing β-galactosidase (blue-stained cells) from the total number of primary human lung fibroblasts (B) and primary human ATII cells (C). Results are expressed as mean ± SE of *n* = 3 independent experiments. Two-way ANOVA was followed by the post hoc Bonferroni test. ^*^ *P* < 0.05 vs. control; ^#^ *P* < 0.05 vs. TGFβ1.

TGF-β1 has demonstrated to promote cell senescence in ATII cells and lung fibroblasts, as well as, to induce cell proliferation in lung fibroblasts [28–30]. In this context, we observed that TGF-β1 at a dose of 10 ng/ml increased lung fibroblast proliferation during 48 h of stimulation (Figure 7A). At this point, lung fibroblasts showed increased β-galactosidase activity, thus indicating a change from active proliferative to senescent fibroblasts (Figure 7B, 7D). ATII cells also acquired secenescent phenotypes after 72 h of TGF-β1 stimulation at a dose of 10 ng/ml (Figure 7C, 7E). Both phenoypes, proliferative and senescent, were inhibited by pirfenidone.

## DISCUSSION

The present study demonstrated novel evidence of the inhibitory effect of pirfenidone on the recently reported intracellular bioactivation of MUC1-CT in IPF. It may be of value to understand the molecular mechanism of action of pirfenidone in IPF, as well as to further develop anti-fibrotic IPF therapies.

**Figure 8:**
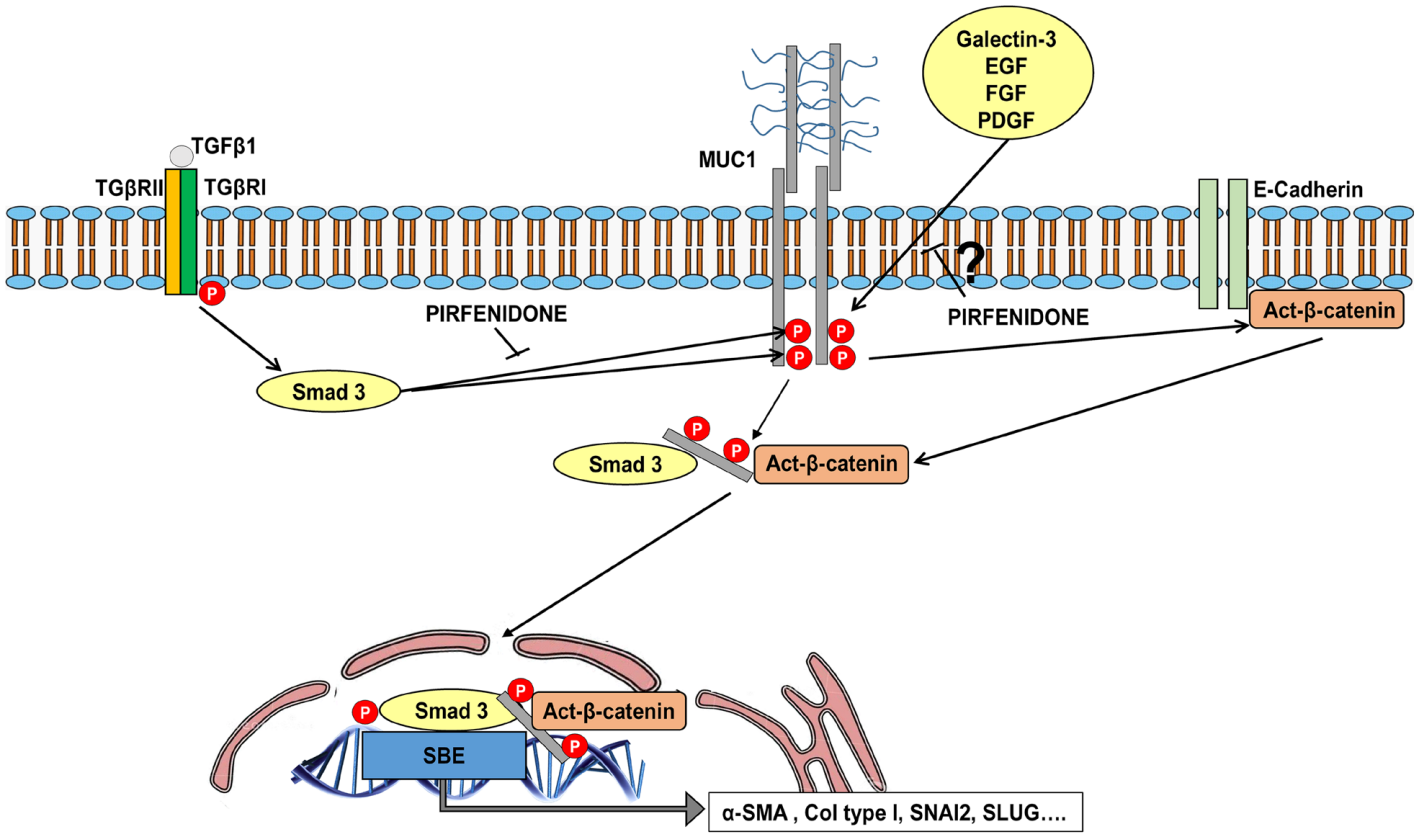
Schematic illustration showing novel evidence for inhibitory effect of pirfenidone on MUC1-CT bioactivation in IPF. TGF-β1 binds to TbRI/II, leading to the recruitment and phosphorylation of SMAD3. Phosphorylated SMAD3 promotes the phosphorylation of MUC1-CT at Thr^41^ and Tyr^46^, thus increasing the active form of β-catenin. MUC1-CT forms protein complexes with phospho (p)-SMAD3 and active (act)-β-catenin in response to TGF-β1; this stimulus promotes nuclear translocation of the phospho-SMAD3/MUC1-CT/act-β-catenin complex, which is required to activate the SMAD-binding element (SBE) DNA sequence and in turn promote pro-fibrotic gene expression, proliferation or cell senescence. Pirfenidone inhibits the TGF-β1-induced Thr^41^ (1224) and Tyr^46^ (1229) MUC1-CT phosphorylations, thus reducing the amount of act-β-catenin. Therefore, the nuclear translocation of the p-SMAD3/MUC1-CT/act-β-catenin complex and induction of SBE is also inhibited by pirfenidone. Additionally, we suggest that pirfenidone also inhibits the MUC1-CT phosphorylation induced by galectin-3 and growth factors such as epidermal growth factor (EGF), fibroblast growth factor (FGF) and platelet derived growth factor (PDGF).

Recently, we found that MUC1-CT was increased in lung tissue from IPF patients, but not in the lungs of healthy subjects, and located mostly in hyperplasic ATII cells and fibroblasts in fibrotic areas. We also found that canonical TGF-β1 induced SMAD3 phosphorylation bioactivated MUC1-CT by phosphorylation at Thr41 (1224) and Tyr46 (1229), thus increasing the activation of β-catenin to form a nuclear complex of phospho-SMAD3/MUC1-CT and MUC1-CT/β-catenin. This nuclear complex was suggested to activate the fibrotic processes of ATII and fibroblast to myofibroblast transitions, cell senescence and fibroblast proliferation through the SBE DNA activation [19] (Figure 8).

Pirfenidone and nintedanib are the two medications approved for the treatment of IPF. Nevertheless, these drugs demonstrate only limitation of the disease course, but they are not able to reverse IPF progression. The mechanism of action of nintedanib is currently well characterized, but the target of pirfenidone is still not well understood [31]. Pirfenidone has demonstrated to increase progression-free survival, to decrease the rate of forced vital capacity (FVC) decline, and to decrease acute exacerbations of IPF patients [32, 33]. Initially, pirfenidone was considered as an antioxidant therapy since it demonstrated O_2_^-^ scavenging activity [34, 35]. However, further studies demonstrated that pirfenidone has a number of anti-inflammatory and anti-fibrotic effects, including downregulation of fibrotic genes in ATII cells, inhibition of collagen synthesis, down-regulation of TGF-β1 cellular effects and reduction of fibroblast proliferation and fibrosis in bleomycin-induced animal models [22, 27]. This anti-fibrotic activity is exhibited not only in the lung, but further in kidney, hepatic, and cardiac fibrosis [22].

Currently, there are no potential data regarding the molecular mechanism of pirfenidone on the TGF-β1 canonical and non-canonical pathways influencing IPF. In this work, we provided a potential mechanism that explain, almost in part, pirfenidone anti-fibrotic effects. Pirfenidone inhibited the TGFβ1-induced MUC1-CT Thr^41^ (1224) and Tyr^46^ (1229) phosphorylations, thus reducing the amount of active-β-catenin and the nuclear translocation of the phospho-SMAD3/MUC1-CT/β-catenin complex and SBE activation (Figure 8).

The major signalling pathway of TGF-β1 is through its TGF-β receptor 1 and 2, activating the cytoplasmic SMAD2 and 3, which act as transcriptional factors to regulate proliferation and fibrotic genes. In this study, we demonstrated that pirfenidone treatment at concentrations of 50µM (~9.2 µg/ml) reduced the TGF-β1-induced SMAD3 nuclear accumulation in ATII cells from healthy donors and primary lung fibroblasts from IPF patients, as well as, the SBE DNA activation in A549 cells. However, pirfenidone did not inhibit SMAD3 phosphorylation in A549 and MRC5 cells. Different results were previously observed in primary human lung fibroblasts, in which pirfenidone reduced the TGF-β-induced SMAD3 phosphorylation [36]. Nevertheless, SMAD3 phosphorylation was only inhibited by the most elevated concentration of pirfenidone (300 µg/ml), which is referred to as supratherapeuthic concentration since it is out of the range of pirfenidone’s serum concentrations (1.64 µg /ml (C_min_) - 14.7 µg/ml (C_max_)) following an oral daily dose of 2403 mg (food and drug administration (FDA) technical pirfenidone brochure). In contrast, Choi *et al*. also used supratherapeuthic concentrations of pirfenidone (500 µg/ml), but similarly to our results, pirfenidone did not inhibit the TGF-β1-induced SMAD3 phosphorylation, but prevented the nuclear accumulation of SMAD3 in the human epithelial cell line ARPE-19 [26]. Additionally, IPF patients treated with pirfenidone tended to have higher levels of p-SMAD than untreated controls [37]. TGF-β1 also propagates its signalling by other signalling pathways such as Wnt/β-catenin pathway or ERK1/2 signalling pathway. In this study, TGF-β1-induced β-catenin activation was inhibited by pirfenidone action in A549 and MRC5 cells. Furthermore, we recently found that the TGF-β1-induced active form of β-catenin was inhibited in siRNA-MUC1 A549 and MRC5 cells, thus suggesting that Thr^41^ (1224) and Tyr^46^ (1229) MUC1-CT phosphorylations are needed to increase active-β-catenin in ATII cells and lung fibroblasts from IPF patients [19]. Therefore, we suggest that pirfenidone inhibits TGF-β1-induced β-catenin activation through inhibition of the MUC1-CT intracellular bioactivation. However, we have not elucidated if pirfenidone has also inhibitory action on Wnt-induced β-catenin activation, although we could hypothesize it since it was previously reported by other authors [38]. In contrast, we observed that TGF-β1-induced ERK1/2 phosphorylation was not inhibited by pirfenidone. Differently, pirfenidone was able to inhibit the activation of the mitogen-activated protein (MAP) kinase signalling in the TGF-β1-treated human renal proximal tubular epithelial cell line HK-2 [39], although supratherapeutic concentrations of pirfenidone (200 and 500 mg/ml) were again used. However, in other study, supratherapeuthic concentrations of pirfenidone had no effect on TGF-β1-induced ERK1/2 phosphorylation in ARPE-19 cells [26].

Previous studies have demonstrated that Thr^41^(1224) and Tyr^46^(1229) MUC1-CT phosphorylations induce binding of the MUC1-CT to β-catenin, thereby promoting the activation of fibrotic genes such as Wnt target genes [40] and CTGF [41]. Furthermore, Thr^41^(1224) and Tyr^46^(1229) phosphorylations promote nuclear translocation of the protein complex MUC1-CT/β-catenin, thus activating target genes [16]. Recently, we found using immunoprecipitation and immunofluorescence studies that MUC1-CT forms nuclear protein complexes with phospho-SMAD3 and active-β-catenin in response to TGF-β1, thus promoting the activation of the SBE DNA sequence to promote pro-fibrotic gene expression [19]. In this study it was demonstrated that pirfenidone inhibited the formation of these complexes and the nuclear translocation of them, thus inhibiting the activation of the SBE DNA sequence.

Lung cells in IPF undergo different types of transformations, such as the ATII to mesenchymal transition or the transformation of lung resident fibroblasts to myofibroblasts [5]. TGF-β1 is considered as the most characterised and most potent cellular transformer of fibroblasts and ATII cells, and multiple pathways can modulate its function [8]. Probably, these phenotypic similarities between ATII cells and fibroblasts upon TGF-β1 action could explain the similar reported pirfenidone’s effect on both types of cells along this study. This study provided evidence of the inhibitory effect of pirfenidone on the TGF-β1-induced ATII to mesenchymal and fibroblast to myofibroblast transitions, as determined by reducing expression of α-SMA and type I collagen in ATII cells and fibroblasts and increasing expression of E-cadherin in ATII cells. In previous studies, it was observed that pirfenidone mediated the inhibition of TGF-β1-induced collagen type I and α-SMA in epithelial cells [23, 39, 42, 43] and fibroblasts [26]. However, some contradictory findings have also been reported in the literature, as pirfenidone did not attenuate the TGF-β1-induced downregulation of E-cadherin in some *in vitro* cancer models [42, 43], but up-regulated the protein expression of E-cadherin in lung tissues of a rat silicosis model [44]. We recently observed that neither A549 cells nor MRC5 fibroblasts transiently transfected with siRNA-MUC1 were transformed to myofibroblasts after TGF-β1 stimulation, as well as, we found using GO-201 that the effects of MUC1-CT on the ATII to mesenchymal and fibroblast to myofibroblast transitions were dependent on MUC1-CT nuclear translocation [19]. Thus, it is suggested that the inhibitory effect of pirfenidone inhibiting MUC1-CT bioactivation and nuclear translocation is the responsible for the inhibitory effect of pirfenidone on the TGF-β1-induced ATII to mesenchymal and fibroblast to myofibroblast transitions.

Senescence of ATII cells and fibroblasts are also key pathological processes in IPF. Furthermore, the senescent fibroblast phenotype co-exists with the proliferative phenotype. Senescent ATII cells and fibroblast are characterized by a metabolically active, hypersecretory and apoptosis-resistant phenotype in the lungs of IPF patients, contributing to the release of fibrotic growth factors, which promote the IPF progression [45–47]. Previously, we reported that the inhibition of MUC1-CT expression via siRNA-MUC1, or of MUC1-CT nuclear translocation using GO-201, blocked the ATII cell senescence, fibroblast senescence and lung fibroblast proliferation induced by TGF-β1 [19]. In this study, pirfenidone also attenuated *in vitro* the TGF-β1-induced fibroblast proliferation and senescence, as well as, the TGF-β1-induced ATII senescence.

Although β-galactosidase staining has been used as the only marker of cell senescence in this study, this is a preliminary screening tool that does not provide confirmation of senescence. Therefore, alternative cellular senescence readouts, such as p21 expression should be addressed to further confirm the effect of pirfenidone on TGF-β1-induced cell senescence.

The inhibition of senescence by pirfenidone could be explained by the inhibition of β-catenin activation, as previous studies have suggested that Wnt/β-catenin TGF-β1 non-canonical pathway acts as a driver of cell senescence in IPF [48], and by inhibition of SBE activation, as activation of this DNA element response also increases the expression of the senescence markers, p21 and p16 [49]. Nevertheless, it has been observed no difference in expression of p21 or p16 in the lungs of untreated or pirfenidone-treated IPF patients [37]. Regarding proliferation, ERK1/2 pathway has demonstrated to mediate the proliferation in cancer cells [50]. However, in this study, pirfenidone did not attenuate the ERK1/2 phosphorylation. Therefore, it is suggested that pirfenidone anti-proliferative action may be through the inhibition of the SBE activation and expression of cyclin kinases [51].

Galectin-3, which is a recognised pro-fibrotic factor in IPF [52], has als0 demonstrated to phosphorylate MUC1-CT, but on a TGF-β1-independent pathway [19]. Therefore, we suggest that pirfenidone could inhibit MUC1-CT bioactivation and fibrotic processes induced or not by TGF-β1. Furthermore, several growth factor receptors, including epidermal growth factor receptor (EGFR), fibroblast growth factor receptor (FGFR)3, platelet-derived growth factor receptor (PDGFR) B and MET, also results in phosphorylation of MUC1-CT [53]. Interestingly, these growth factor receptors not only participate in IPF, but also in other proliferative diseases such as several malignancies, thus suggesting a protective role of pirfenidone for all of them. Indeed, pirfenidone has demonstrated anti-neoplastic effects in preclinical studies [54], as well as, it has been observed that the incidence of lung cancer in IPF patients treated with pirfenidone is significantly lower than in a non-pirfenidone IPF patient group [55].

In summary, the present study provided novel evidence of pirfenidone’s inhibitory effect on TGF-β1-induced fibrotic cellular processes, through the inhibitory effects of pirfenidone on MUC1-CT phosphorylations, β-catenin activation and formation of the nuclear complex of phospho-SMAD3/MUC1-CT/act-β-catenin and the following SBE activation.

## MATERIALS AND METHODS

### Isolation and culture of human alveolar type II cells and lung fibroblast

Primary ATII cells were obtained from lung parenchyma of control donors. Lung parenchyma tissue was cut in approximately 1 mm thick sections and lavaged with saline. The lung sections were digested with dispase II (final concentration, 20 U/100 ml), collagenase/dispase (final concentration, 10 mg/100 ml) and DNase I (final concentration, 1 mg/ 100 ml) at 37°C for 120 minutes. After digestion, the lung sections were filtered through nylon meshes ranging in pore size from 100 to 20 mm. The resulting cell suspension was centrifuged (300 × g, 20 min at 15°C) through a sterile Percoll gradient and the alveolar type II cell–rich band was collected. If it was required, ATII cell-rich band was resuspended in 5 ml of red blood cell lysis buffer and recovered as a pellet by centrifugation at 300 × g for 10 minutes. Finally, cells were resuspended in a buffer containing phosphate buffered saline (PBS) pH 7.2, 2mM ethylenediaminetetraacectic acid (EDTA) and 0.5% bovine serum albumin (BSA). ATII cells were negatively separated using CD45 microbeads in a magnetic field (QuadroMACS™ Separator. Miltenyi Biotec). Total cells were counted to establish the final yield of freshly purified cells. Alveolar type II cell viability was assessed with trypan blue (Sigma), showing greater than 95% viability. Cell purity was routinely assessed by epithelial cell morphology and immunofluorescence analysis with pan-cytokeratin and pro-surfactant protein C (both positive) as well as α-SMA and CD45 (both negative) of cytocentrifuge preparations of ATII cells. ATII cells used throughout this study demonstrated 95% ± 3% purity. Finally, ATII cells were suspended in Dulbecco's Modified Eagle's Medium (DMEM) plus 10% FCS, 2 mM l-glutamine, 100 U/ml penicillin, and 100 g/ml streptomycin and cultured for 48 hours to allow attachment. Phenotypic characterization was done after this time period. After media change, cells were cultured for a maximum of 3 days in a humidified atmosphere of 5% CO2 at 37°C. The bronchoalveolar A549 cell line has been used to model ATII behavior. Although A549 cell line can show different properties than primary ATII cells, we have used A549 cells for some *in vitro* experiments because their stability and the possibility to obtain enough quantity of protein to explore cellular mechanistic pathways. The A549 human alveolar type II cell line was purchased from American Type Culture Collection (Rockville, MD, USA), which authenticates their lines via short tandem repeat profiling. They were cultured in supplemented Roswell Park Memorial Institute (RPMI) 1640 medium at 37°C in a humidified atmosphere of 5% CO2 in air, as outlined [56]. Cells at 60–70% confluence were serum-deprived by incubation for 12–18 h in RPMI 1640 medium containing 0.1% (v/v) foetal bovine serum prior to stimulation with TGFβ1 or other agents.

Primary human lung fibroblasts were obtained from lung parenchyma of macroscopically fibrotic affected areas of IPF patients as previously outlined [57]. Lung parenchyma was cut into small pieces, treated with pronase (1 mg/mL; Calbiochem^®^, Novabiochem^®^, San Diego, CA, USA) at 37°C for 30 min, placed in cell culture plates and incubated in DMEM supplemented with 10% foetal calf serum (Sigma, St. Louis, MO, USA), 100 U/mL penicillin/streptomycin and 2% fungizone (GIBCO, Grand Island, NY, USA). After approximately 2 weeks, fibroblasts had grown from the tissue and were passaged by standard trypsinisation. Cells from passages 3–10 were used in all experiments described in the present study.

Normal lung fibroblast MRC5 was purchased from American Type Culture Collection (Rockville, MD, USA) and were cultured in 10% FCS supplemented RPMI-1640 medium at 37°C in a humidified atmosphere of 5% CO2 in air.

For *in vitro* studies, ATII/A549 or primary lung fibroblast/ MRC5 were stimulated with recombinant TGF-β1 (Sigma Aldrich; catalog no. T7039) for the indicated times and concentrations, replacing culture medium and stimulus every 24 h. TGFβ1 has demonstrated to induce cell phenotypic changes such as epithelial to mesenchymal transition at the indicated concentrations [58].

Pirfenidone (antifibrotic drug approved for the IPF treatment; Sigma Aldrich; catalog no. G5170) was added 30 min before stimulus and remained together with the stimulus until experimental evaluation. For all the experiments, 50 µM doses of pirfenidone were used. This concentration was selected since 50 µM (9 mg/ml) is between the C_max_ (14.7 mg/ml) and C_min_ (1.64 mg/ml) serum concentrations following a daily dose of 2403 mg of pirfenidone (FDA technical pirfenidone brochure). It did not affect cell viability assessed with trypan blue (Sigma), showing greater than 95% viability.

### Western blotting analysis

Western blotting analysis was used to detect changes in ATII/A549 and lung fibroblast/MRC5 cell protein expression. Cells were scraped from a confluent 25-cm^2^ flask and lysed on ice with a lysis buffer comprising a complete inhibitor cocktail plus 1 mM ethylenediaminetetraacectic acid (Roche Diagnostics Ltd., West Sussex, UK) with 20 mM Tris base, 0.9% NaCl, 0.1% Triton X-100, 1 mM dithiothreitol, and 1 mg/mL pepstatin A. The Bio-Rad assay (Bio-Rad Laboratories Ltd., Herts, UK) was used according to the manufacturer’s instructions to quantify the level of protein in each sample to ensure equal protein loading. Sodium dodecyl sulfate polyacrylamide gel electrophoresis was used to separate the proteins according to their molecular weight. Briefly, 15 µg of proteins (denatured) along with a molecular weight protein marker (Bio-Rad Kaleidoscope marker; Bio-Rad Laboratories) were loaded onto an acrylamide gel consisting of a 5% acrylamide stacking gel stacked on top of a 10% acrylamide resolving gel and run through the gel by application of 100 V for 1 h. Proteins were transferred from the gel to a polyvinylidene difluoride membrane using a wet-blotting method. The membrane was blocked with 5% Marvel in PBS containing 0.1% Tween20 (PBS-T), probed with the following antibodies: alpha smooth muscle actin (α-SMA) (1:1000) antibody (42 KDa, monoclonal antibody, Sigma Aldrich, Madrid, Spain; catalog no. A5228), collagen type I (1:1000) antibody (139 KDa, polyclonal antibody; Calbiochem Darmstadt, Germany; catalog no. 234167), E-cadherin (1:1000) antibody (120 KDa, monoclonal antibody; ECM BioScience, Versailles, USA; catalog no. CM1681), phospho (p)-SMAD3 (1:1000) antibody (50 KDa, monoclonal antibody; Millipore, Madrid, Spain; catalog no. PS1023), MUC1-P/T-1224 (1:500) antibody (122 KDa, Abgent, California, USA; catalog no. AP3728a), MUC1-P/Y-1229 (1:500) antibody (120 KDa, Abcam, Cambridge, UK; catalog no. ab79226), non-phospho (active) β-catenin (Ser33/37/Thr41) (1:500) antibody (96 KDa, monoclonal antibody; Cell Signaling; catalogue no. 8814S), phospho-ERK1/2 (1:1000) antibody (42 and 44 KDa, monoclonal antibody; Sigma; cat. n°: M-9692), and normalized to total anti-human β-actin (1:1000) antibody (42 KDa, monoclonal antibody, catalog no. A1978; Sigma), total anti-Smad3 (1:1000) antibody (48 KDa, polyclonal antibody; Calbiochem, Madrid, Spain; catalog no. 566414), total MUC1-CT (1:500) antibody (21 KDa, Novus Biologicals, Colorado, USA; catalog no. NBP1-60046), total anti-β-catenin antibody (92 KDa, polyclonal antibody; Cell Signaling; catalogue no. 9562S) and total ERK1/2 (1:1,000) antibody (44 KDa, monoclonal antibody; Cell Signalling, Boston, Massachusetts, USA; catalogue no. 4695). The enhanced chemiluminescence method of protein detection using enhanced chemiluminescence reagents (ECL Plus; Amersham GE Healthcare, Buckinghamshire, UK) was used to detect labeled proteins. Densitometry of films was performed using the Image J 1.42q software (available at http://rsb.info.nih.gov/ij/, USA). Results of target protein expression are expressed as the percentage of the densitometry of the endogenous controls β-actin, total SMAD3, total MUC1-CT, total ERK1/2 or total β-catenin, as appropriate.

### Real-time RT-PCR

Total RNA was isolated using TriPure^®^ Isolation Reagent (Roche, Indianapolis, USA). The integrity of the extracted RNA was confirmed with Bioanalyzer (Agilent, Palo Alto, CA, USA). Reverse transcription was performed in 300 ng of total RNA with a TaqMan reverse transcription reagents kit (Applied Biosystems, Perkin-Elmer Corporation, CA, USA). cDNA was amplified with specific primers and probes predesigned by Applied Biosystems for MUC1 (Hs00159357_m1), α-SMA (Hs00559403_m1), α_1_(I)-collagen (collagen type I; Hs00164004_m1), SNAIL (Hs00195591_m1), SNAI2 (Hs00161904_m1) and E-cadherin (Hs01023894_m1) in a 7900HT Fast Real-Time PCR System (Applied Biosystems) using Universal Master Mix (Applied Biosystems). Expression of the target gene was expressed as the fold increase or decrease relative to the expression of β-actin as an endogenous control (Applied Biosystems; Hs01060665). The mean value of the replicates for each sample was calculated and expressed as the cycle threshold (Ct). The level of gene expression was then calculated as the difference (∆Ct) between the Ct value of the target gene and the Ct value of β-actin. The fold changes in the target gene mRNA levels were designated 2^-∆Ct^.

### Proliferation

Fibroblast proliferation was measured by colorimetric immunoassay based on BrdU incorporation during DNA synthesis using a cell proliferation enzyme-linked immunosorbent assay BrdU kit (Roche, Mannheim, Germany) according to the manufacturer’s protocol. Cells were seeded at a density of 3 × 10^3^ cells/well on 96-well plates and incubated for 24 hours. TGF-β1 stimulation was incubated for 48 h. The 490 nm absorbance was quantified using a microplate spectrophotometer (Victor 1420 Multilabel Counter, PerkinElmer). Proliferation data refer to the absorbance values of BrdU-labeled cellular DNA content per well.

### Cell senescence

Cells were stimulated with TGF-β1 10 ng/ml during 72h. The senescence cell histochemical staining kit (Sigma Aldrich; catalogue no. CS0030) based on a histochemical staining for β-galactosidase activity was used. After TGF-β1 stimulation, cells were fixed with the fixation buffer provided by the kit (solution containing 20% formaldehyde, 2% glutaraldehyde, 70.4 mM Na2HPO4, 14.7 mM KH2PO4, 1.37 M NaCl, and 26.8 mM KCl) for 6–7 minutes at room temperature. Cells were stained with the staining mixture provided by the kit and incubated at 37°C without CO_2_ overnight. Finally, cells were observed under a microscope to count the blue-stained cells and the total number of cells x field. Results were expressed as % senescence (β-galactosidase blue positive cells) relative to the total number of cells in each field.

### SBE assay

The SBE Reporter kit (Cat#: 60654, BPSBioscience) was used for monitoring the activity of TGF-β/SMAD signaling pathway in the cultured cells. One day before transfection, cells were seed at a density of 2 × 10^4^ cells per well in 200 µl of 10% fetal bovine serum (FBS) medium (antibiotic-free) so that cells were 70% confluent at the time of SBE reporter transfection. Plate was incubated at 37°C in a CO_2_ incubator. Next day, the following complexes were prepared: 1 µg of SBE luciferase reporter vector + constitutively expressing renilla luciferase vector diluted in 22.5 µl of Opti-MEM I medium (antibiotic-free). The control transfection was the non-inducible luciferase vector + constitutively expressing renilla luciferase vector. Complexes were incubated for 5 minutes at room temperature. Diluted DNA was combined with diluted Lipofectamine 2000 and incubated for 25 minutes at room temperature. 45 µl of complexes were added to each well containing cells and medium and cells were incubated at 37°C in a 5% CO_2_ incubator during 24 hours. After 24 hours, medium was changed to 0.5% FBS fresh medium and TGF-β1 (final concentration 10 ng/ml) was added to stimulate wells. After 18 hours of stimulation, luciferase activity was evaluated by dual-luciferase reporter assay system (Promega, catalogue no. E1910) following manufacturer’s protocol. To obtain the normalized luciferase activity of the SBE reporter, background was subtracted, and the ratio of firefly luminescence from the SBE reporter to renilla luminescence from the control renilla luciferase vector was represented.

### Immunoprecipitation

Equal amounts of protein (200 µg) from total protein extracts were incubated with 2 µg of p-Smad3, act-β-catenin or anti-MUC1-CT antibodies and the IgG isotype control. The immune complexes were precipitated with protein G on Sepharose 4B fast flow beads (Sigma Aldrich; catalogue no. P-3296) overnight at 4°C. After washing three times with NET buffer containing 50 mM Tris-HCl at pH 8.0, 150 mM NaCl, and 0.1% Nonidet P-40, the bound materials were eluted from the immunoprecipitates in reducing SDS-PAGE loading buffer containing 10% SDS, 1 M Tris-HCl at pH 6.8, 50% glycerol, 10% 2-mercaptoethanol, and 2% bromophenol blue at 100°C for 10 min. Immunoprecipitated protein complexes were assayed by western blotting as described above and probed using p-SMAD3, act-β-catenin or anti-MUC1-CT antibodies, as appropriate.

### Immunofluorescence

MUC1-CT, p-SMAD3 and act-β-catenin in ATII and fibroblasts were analyzed by immunofluorescence. Cells were firstly fixed in paraformaldehyde (4%) for 24 h. Following it, cells were permeabilized (20 mM HEPES at pH 7.6, 300 mM sucrose, 50 mM NaCl, 3 mM MgCl_2_, 0.5% Triton X-100), blocked (10% goat serum in PBS), and incubated with the primary antibodies overnight at 4°C, followed by secondary antibody anti-rabbit/mouse rhodamine/FITC- (1:100, Molecular Probes). Colocalization of MUC1-CT/ p-SMAD3 and MUC1-CT/ act-β-catenin was performed using a confocal spectral Leica TCS SP2 microscope with ×1000 magnification and 3× zoom. Red (HeNe 543 nm), green (HeNe 488 nm), and blue (Ar 351 nm, 364 nm) lasers were used. Colocalization studies were performed using the Leica confocal software v2.61. The cell images with colocalized points of the two laser canals were transformed into a white color.

### Statistical analysis

Statistical analysis of results was carried out by parametric analysis. *P* < 0.05 was considered statistically significant. Results were expressed as mean ± SE of n experiments. Multiple comparisons were analysed by one-way or two-way analysis of variance (ANOVA) followed by Bonferroni post hoc test. A *P* value < 0.05 was considered significant.

## Abbreviations

ATII: alveolar type II epithelial cells
CT: cytoplasmic tail
ECM: extracellular matrix
FBS: fetal bovine serum
FDA: food and drug administration
IPF: Idiopathic pulmonary fibrosis
MUC1: mucin 1
PBS: phosphate buffered saline
SBE: SMAD-binding element
TGF-β1: transforming growth factor-β1

## References

[1] Fernandez Fabrellas E , Peris Sanchez R , Sabater Abad C , Juan Samper G . Prognosis and Follow-Up of Idiopathic Pulmonary Fibrosis. Med Sci. 2018; 6. 10.3390/medsci6020051. 29904028 PMC6024649

[2] Bellaye PS , Kolb M . Why do patients get idiopathic pulmonary fibrosis? Current concepts in the pathogenesis of pulmonary fibrosis. BMC Med. 2015; 13:176. 10.1186/s12916-015-0412-6. 26400687 PMC4581472

[3] Singh SR , Hall IP . Airway myofibroblasts and their relationship with airway myocytes and fibroblasts. Proc Am Thorac Soc. 2008; 5:127–132. 10.1513/pats.200706-070VS. 18094095

[4] Thannickal VJ , Toews GB , White ES , Lynch JP 3rd , Martinez FJ . Mechanisms of pulmonary fibrosis. Annu Rev Med. 2004; 55:395–417. 10.1146/annurev.med.55.091902.103810. 14746528

[5] Bagnato G , Harari S . Cellular interactions in the pathogenesis of interstitial lung diseases. Eur Respir Rev. 2015; 24:102–114. 10.1183/09059180.00003214. 25726561 PMC9487765

[6] Schafer MJ , White TA , Iijima K , Haak AJ , Ligresti G , Atkinson EJ , Oberg AL , Birch J , Salmonowicz H , Zhu Y , Mazula DL , Brooks RW , Fuhrmann-Stroissnigg H , et al. Cellular senescence mediates fibrotic pulmonary disease. Nat Commun. 2017; 8:14532. 10.1038/ncomms14532. 28230051 PMC5331226

[7] Selman M , Pardo A . Revealing the pathogenic and aging-related mechanisms of the enigmatic idiopathic pulmonary fibrosis. an integral model. Am J Respir Crit Care Med. 2014; 189:1161–1172. 10.1164/rccm.201312-2221PP. 24641682

[8] Akhurst RJ , Hata A . Targeting the TGFbeta signalling pathway in disease. Nat Rev Drug Discov. 2012; 11:790–811. 10.1038/nrd3810. 23000686 PMC3520610

[9] Blobe GC , Schiemann WP , Lodish HF . Role of transforming growth factor beta in human disease. N Engl J Med. 2000; 342:1350–1358. 10.1056/NEJM200005043421807. 10793168

[10] Bonniaud P , Margetts PJ , Ask K , Flanders K , Gauldie J , Kolb M . TGF-beta and Smad3 signaling link inflammation to chronic fibrogenesis. J Immunol. 2005; 175:5390–5395. 10.4049/jimmunol.175.8.5390. 16210645

[11] Selman M , Pardo A , Kaminski N . Idiopathic pulmonary fibrosis: aberrant recapitulation of developmental programs? PLoS Med. 2008; 5:e62. 10.1371/journal.pmed.0050062. 18318599 PMC2265304

[12] Baarsma HA , Konigshoff M . ‘WNT-er is coming’: WNT signalling in chronic lung diseases. Thorax. 2017; 72:746–759. 10.1136/thoraxjnl-2016-209753. 28416592 PMC5537530

[13] Ishikawa N , Hattori N , Yokoyama A , Kohno N . Utility of KL-6/MUC1 in the clinical management of interstitial lung diseases. Respir Investig. 2012; 50:3–13. 10.1016/j.resinv.2012.02.001. 22554854

[14] Palmai-Pallag T , Khodabukus N , Kinarsky L , Leir SH , Sherman S , Hollingsworth MA , Harris A . The role of the SEA (sea urchin sperm protein, enterokinase and agrin) module in cleavage of membrane-tethered mucins. FEBS J. 2005; 272:2901–2911. 10.1111/j.1742-4658.2005.04711.x. 15943821

[15] Wakamatsu K , Nagata N , Kumazoe H , Oda K , Ishimoto H , Yoshimi M , Takata S , Hamada M , Koreeda Y , Takakura K , Ishizu M , Hara M , Ise S , et al. Prognostic value of serial serum KL-6 measurements in patients with idiopathic pulmonary fibrosis. Respir Investig. 2017; 55:16–23. 10.1016/j.resinv.2016.09.003. 28012488

[16] Hattrup CL , Gendler SJ . Structure and function of the cell surface (tethered) mucins. Annu Rev Physiol. 2008; 70:431–457. 10.1146/annurev.physiol.70.113006.100659. 17850209

[17] Bafna S , Kaur S , Batra SK . Membrane-bound mucins: the mechanistic basis for alterations in the growth and survival of cancer cells. Oncogene. 2010; 29:2893–2904. 10.1038/onc.2010.87. 20348949 PMC2879972

[18] van Putten JPM , Strijbis K . Transmembrane Mucins: Signaling Receptors at the Intersection of Inflammation and Cancer. J Innate Immun. 2017; 9:281–299. 10.1159/000453594. 28052300 PMC5516414

[19] Milara J , Ballester B , Montero P , Escriva J , Artigues E , Alos M , Pastor-Clerigues A , Morcillo E , Cortijo J . MUC1 intracellular bioactivation mediates lung fibrosis. Thorax. 2020; 75:132–142. 10.1136/thoraxjnl-2018-212735. 31801904

[20] Noble PW , Albera C , Bradford WZ , Costabel U , Glassberg MK , Kardatzke D , King TE Jr , Lancaster L , Sahn SA , Szwarcberg J , Valeyre D , du Bois RM , Group CS . Pirfenidone in patients with idiopathic pulmonary fibrosis (CAPACITY): two randomised trials. Lancet. 2011; 377:1760–1769. 10.1016/S0140-6736(11)60405-4. 21571362

[21] Datta A , Scotton CJ , Chambers RC . Novel therapeutic approaches for pulmonary fibrosis. Br J Pharmacol. 2011; 163:141–172. 10.1111/j.1476-5381.2011.01247.x. 21265830 PMC3085875

[22] Schaefer CJ , Ruhrmund DW , Pan L , Seiwert SD , Kossen K . Antifibrotic activities of pirfenidone in animal models. Eur Respir Rev. 2011; 20:85–97. 10.1183/09059180.00001111. 21632796 PMC9487788

[23] Hisatomi K , Mukae H , Sakamoto N , Ishimatsu Y , Kakugawa T , Hara S , Fujita H , Nakamichi S , Oku H , Urata Y , Kubota H , Nagata K , Kohno S . Pirfenidone inhibits TGF-beta1-induced over-expression of collagen type I and heat shock protein 47 in A549 cells. BMC Pulm Med. 2012; 12:24. 10.1186/1471-2466-12-24. 22694981 PMC3403980

[24] Lin X , Yu M , Wu K , Yuan H , Zhong H . Effects of pirfenidone on proliferation, migration, and collagen contraction of human Tenon’s fibroblasts *in vitro* . Invest Ophthalmol Vis Sci. 2009; 50:3763–3770. 10.1167/iovs.08-2815. 19264889

[25] Oku H , Shimizu T , Kawabata T , Nagira M , Hikita I , Ueyama A , Matsushima S , Torii M , Arimura A . Antifibrotic action of pirfenidone and prednisolone: different effects on pulmonary cytokines and growth factors in bleomycin-induced murine pulmonary fibrosis. Eur J Pharmacol. 2008; 590:400–408. 10.1016/j.ejphar.2008.06.046. 18598692

[26] Choi K , Lee K , Ryu SW , Im M , Kook KH , Choi C . Pirfenidone inhibits transforming growth factor-beta1-induced fibrogenesis by blocking nuclear translocation of Smads in human retinal pigment epithelial cell line ARPE-19. Mol Vis. 2012; 18:1010–1020. 22550395 PMC3339036

[27] Lehmann M , Buhl L , Alsafadi HN , Klee S , Hermann S , Mutze K , Ota C , Lindner M , Behr J , Hilgendorff A , Wagner DE , Konigshoff M . Differential effects of Nintedanib and Pirfenidone on lung alveolar epithelial cell function in *ex vivo* murine and human lung tissue cultures of pulmonary fibrosis. Respir Res. 2018; 19:175. 10.1186/s12931-018-0876-y. 30219058 PMC6138909

[28] Alvarez D , Cardenes N , Sellares J , Bueno M , Corey C , Hanumanthu VS , Peng Y , D’Cunha H , Sembrat J , Nouraie M , Shanker S , Caufield C , Shiva S , et al. IPF lung fibroblasts have a senescent phenotype. Am J Physiol Lung Cell Mol Physiol. 2017; 313:L1164–L1173. 10.1152/ajplung.00220.2017. 28860144 PMC6148001

[29] Minagawa S , Araya J , Numata T , Nojiri S , Hara H , Yumino Y , Kawaishi M , Odaka M , Morikawa T , Nishimura SL , Nakayama K , Kuwano K . Accelerated epithelial cell senescence in IPF and the inhibitory role of SIRT6 in TGF-beta-induced senescence of human bronchial epithelial cells. Am J Physiol Lung Cell Mol Physiol. 2011; 300:L391–L401. 10.1152/ajplung.00097.2010. 21224216 PMC3284316

[30] Penke LR , Speth JM , Dommeti VL , White ES , Bergin IL , Peters-Golden M . FOXM1 is a critical driver of lung fibroblast activation and fibrogenesis. J Clin Invest. 2018; 128:2389–2405. 10.1172/JCI87631. 29733296 PMC5983327

[31] Lederer DJ , Martinez FJ . Idiopathic Pulmonary Fibrosis. N Engl J Med. 2018; 378:1811–1823. 10.1056/NEJMra1705751. 29742380

[32] Azuma A , Nukiwa T , Tsuboi E , Suga M , Abe S , Nakata K , Taguchi Y , Nagai S , Itoh H , Ohi M , Sato A , Kudoh S . Double-blind, placebo-controlled trial of pirfenidone in patients with idiopathic pulmonary fibrosis. Am J Respir Crit Care Med. 2005; 171:1040–1047. 10.1164/rccm.200404-571OC. 15665326

[33] Taniguchi H , Ebina M , Kondoh Y , Ogura T , Azuma A , Suga M , Taguchi Y , Takahashi H , Nakata K , Sato A , Takeuchi M , Raghu G , Kudoh S , et al. Pirfenidone in idiopathic pulmonary fibrosis. Eur Respir J. 2010; 35:821–829. 10.1183/09031936.00005209. 19996196

[34] Mitani Y , Sato K , Muramoto Y , Karakawa T , Kitamado M , Iwanaga T , Nabeshima T , Maruyama K , Nakagawa K , Ishida K , Sasamoto K . Superoxide scavenging activity of pirfenidone-iron complex. Biochem Biophys Res Commun. 2008; 372:19–23. 10.1016/j.bbrc.2008.04.093. 18468515

[35] Salazar-Montes A , Ruiz-Corro L , Lopez-Reyes A , Castrejon-Gomez E , Armendariz-Borunda J . Potent antioxidant role of pirfenidone in experimental cirrhosis. Eur J Pharmacol. 2008; 595:69–77. 10.1016/j.ejphar.2008.06.110. 18652820

[36] Conte E , Gili E , Fagone E , Fruciano M , Iemmolo M , Vancheri C . Effect of pirfenidone on proliferation, TGF-beta-induced myofibroblast differentiation and fibrogenic activity of primary human lung fibroblasts. Eur J Pharm Sci. 2014; 58:13–19. 10.1016/j.ejps.2014.02.014. 24613900

[37] Zhang Y , Jones KD , Achtar-Zadeh N , Green G , Kukreja J , Xu B , Wolters PJ . Histopathological and molecular analysis of idiopathic pulmonary fibrosis lungs from patients treated with pirfenidone or nintedanib. Histopathology. 2019; 74:341–349. 10.1111/his.13745. 30152895

[38] Zou WJ , Huang Z , Jiang TP , Shen YP , Zhao AS , Zhou S , Zhang S . Pirfenidone Inhibits Proliferation and Promotes Apoptosis of Hepatocellular Carcinoma Cells by Inhibiting the Wnt/beta-Catenin Signaling Pathway. Med Sci Monit. 2017; 23:6107–6113. 10.12659/MSM.907891. 29276937 PMC5749136

[39] Li Z , Liu X , Wang B , Nie Y , Wen J , Wang Q , Gu C . Pirfenidone suppresses MAPK signalling pathway to reverse epithelial-mesenchymal transition and renal fibrosis. Nephrology (Carlton). 2017; 22:589–597. 10.1111/nep.12831. 27245114

[40] Huang L , Chen D , Liu D , Yin L , Kharbanda S , Kufe D . MUC1 oncoprotein blocks glycogen synthase kinase 3beta-mediated phosphorylation and degradation of beta-catenin. Cancer Res. 2005; 65:10413–10422. 10.1158/0008-5472.CAN-05-2474. 16288032

[41] Behrens ME , Grandgenett PM , Bailey JM , Singh PK , Yi CH , Yu F , Hollingsworth MA . The reactive tumor microenvironment: MUC1 signaling directly reprograms transcription of CTGF. Oncogene. 2010; 29:5667–5677. 10.1038/onc.2010.327. 20697347 PMC3412169

[42] Fujiwara A , Shintani Y , Funaki S , Kawamura T , Kimura T , Minami M , Okumura M . Pirfenidone plays a biphasic role in inhibition of epithelial-mesenchymal transition in non-small cell lung cancer. Lung Cancer. 2017; 106:8–16. 10.1016/j.lungcan.2017.01.006. 28285699

[43] Kurimoto R , Ebata T , Iwasawa S , Ishiwata T , Tada Y , Tatsumi K , Takiguchi Y . Pirfenidone may revert the epithelial-to-mesenchymal transition in human lung adenocarcinoma. Oncol Lett. 2017; 14:944–950. 10.3892/ol.2017.6188. 28693256 PMC5494670

[44] Guo J , Yang Z , Jia Q , Bo C , Shao H , Zhang Z . Pirfenidone inhibits epithelial-mesenchymal transition and pulmonary fibrosis in the rat silicosis model. Toxicol Lett. 2019; 300:59–66. 10.1016/j.toxlet.2018.10.019. 30394303

[45] Waters DW , Blokland KEC , Pathinayake PS , Burgess JK , Mutsaers SE , Prele CM , Schuliga M , Grainge CL , Knight DA . Fibroblast senescence in the pathology of idiopathic pulmonary fibrosis. Am J Physiol Lung Cell Mol Physiol. 2018; 315:L162–L172. 10.1152/ajplung.00037.2018. 29696986 PMC6139657

[46] Lehmann M , Korfei M , Mutze K , Klee S , Skronska-Wasek W , Alsafadi HN , Ota C , Costa R , Schiller HB , Lindner M , Wagner DE , Gunther A , Konigshoff M . Senolytic drugs target alveolar epithelial cell function and attenuate experimental lung fibrosis *ex vivo* . Eur Respir J. 2017; 50. 10.1183/13993003.02367-2016. 28775044 PMC5593348

[47] Rana T , Jiang C , Liu G , Miyata T , Antony V , Thannickal VJ , Liu RM . PAI-1 Regulation of TGF-beta1-induced ATII Cell Senescence, SASP Secretion, and SASP-mediated Activation of Alveolar Macrophages. Am J Respir Cell Mol Biol. 2020; 62:319–330. 10.1165/rcmb.2019-0071OC. 31513752 PMC7055702

[48] Lehmann M , Baarsma HA , Konigshoff M . WNT Signaling in Lung Aging and Disease. Annals of the American Thoracic Society. 2016; 13:S411–S416. 10.1513/AnnalsATS.201608-586AW. 28005418

[49] Carlson ME , Hsu M , Conboy IM . Imbalance between pSmad3 and Notch induces CDK inhibitors in old muscle stem cells. Nature. 2008; 454:528–532. 10.1038/nature07034. 18552838 PMC7761661

[50] Mori Y , Akita K , Yashiro M , Sawada T , Hirakawa K , Murata T , Nakada H . Binding of Galectin-3, a beta-Galactoside-binding Lectin, to MUC1 Protein Enhances Phosphorylation of Extracellular Signal-regulated Kinase 1/2 (ERK1/2) and Akt, Promoting Tumor Cell Malignancy. J Biol Chem. 2015; 290:26125–26140. 10.1074/jbc.M115.651489. 26342075 PMC4646264

[51] Zhu X , Ozturk F , Liu C , Oakley GG , Nawshad A . Transforming growth factor-beta activates c-Myc to promote palatal growth. J Cell Biochem. 2012; 113:3069–3085. 10.1002/jcb.24184. 22573578 PMC3418447

[52] Mackinnon AC , Gibbons MA , Farnworth SL , Leffler H , Nilsson UJ , Delaine T , Simpson AJ , Forbes SJ , Hirani N , Gauldie J , Sethi T . Regulation of transforming growth factor-beta1-driven lung fibrosis by galectin-3. Am J Respir Crit Care Med. 2012; 185:537–546. 10.1164/rccm.201106-0965OC. 22095546 PMC3410728

[53] Kufe DW . Mucins in cancer: function, prognosis and therapy. Nat Rev Cancer. 2009; 9:874–885. 10.1038/nrc2761. 19935676 PMC2951677

[54] Mediavilla-Varela M , Boateng K , Noyes D , Antonia SJ . The anti-fibrotic agent pirfenidone synergizes with cisplatin in killing tumor cells and cancer-associated fibroblasts. BMC Cancer. 2016; 16:176. 10.1186/s12885-016-2162-z. 26935219 PMC4776434

[55] Miura Y , Saito T , Tanaka T , Takoi H , Yatagai Y , Inomata M , Nei T , Saito Y , Gemma A , Azuma A . Reduced incidence of lung cancer in patients with idiopathic pulmonary fibrosis treated with pirfenidone. Respir Investig. 2018; 56:72–79. 10.1016/j.resinv.2017.09.007. 29325685

[56] Mata M , Sarria B , Buenestado A , Cortijo J , Cerda M , Morcillo EJ . Phosphodiesterase 4 inhibition decreases MUC5AC expression induced by epidermal growth factor in human airway epithelial cells. Thorax. 2005; 60:144–152. 10.1136/thx.2004.025692. 15681504 PMC1747298

[57] Milara J , Serrano A , Peiro T , Gavalda A , Miralpeix M , Morcillo EJ , Cortijo J . Aclidinium inhibits human lung fibroblast to myofibroblast transition. Thorax. 2012; 67:229–237. 10.1136/thoraxjnl-2011-200376. 21957094 PMC3282044

[58] Milara J , Navarro R , Juan G , Peiro T , Serrano A , Ramon M , Morcillo E , Cortijo J . Sphingosine-1-phosphate is increased in patients with idiopathic pulmonary fibrosis and mediates epithelial to mesenchymal transition. Thorax. 2012; 67:147–156. 10.1136/thoraxjnl-2011-200026. 22106015

